# Supplementary material for: Outcomes of Nonagenarians with Acute Myocardial Infarction with or without Coronary Intervention
Source: J Clin Med. 2022 Mar 14;11(6):1593. doi: 10.3390/jcm11061593 (PMC8955178; doi:10.3390/jcm11061593)
Supplement: Supplementary file 1 [file jcm-11-01593-s001.zip › Supplemental Table S1 (Table S1) (2022-03-05) (JCM).pdf]

Table S1. Clinical characteristics at the time of hospital visit of participants.

Characteristics	PCI group (n = 320)	No-PCI group (n = 147)	*p*-value
**Age, years**	91.99 ± 2.03	92.14 ± 2.21	0.485
**Atypical anginal pain**	78 (24.5)	68 (46.6)	**<0.001**
**EMS utilization**	61 (19.1)	28 (19.0)	0.997
**Onset-to-door time ≥ 12 h**	82 (25.8)	45 (30.6)	0.277
**Off-hour presentation**	175 (54.7)	83 (56.5)	0.720

Values are presented as number (percentage) for categorical values and means ± standard deviation for continuous variables.

EMS, emergency medical service; PCI, percutaneous coronary intervention.
