# Supplementary material for: Outcomes of Nonagenarians with Acute Myocardial Infarction with or without Coronary Intervention
Source: J Clin Med. 2022 Mar 14;11(6):1593. doi: 10.3390/jcm11061593 (PMC8955178; doi:10.3390/jcm11061593)
Supplement: Supplementary file 1 [file jcm-11-01593-s001.zip › Supplemental Table S2 (Table S2) (2022-03-05) (JCM).pdf]

Table S2. Coronary angiography and procedural characteristics in PCI-treated nonagenarian AMI patients.

Characteristics	PCI group (n = 320)
Transfemoral approach (%)	187 (66.8)
GPIIb/IIIa inhibitor use (%)	26 (8.1)
Thrombus aspiration	47 (14.7)
Infarct-related artery	
LMCA (%)	7 (2.2)
LAD (%)	156 (48.9)
LCX (%)	42 (13.2)
RCA (%)	114 (35.7)
ACC/AHA lesion classification	
Type A or B1 (%)	32 (10.7)
Type B2 or C (%)	268 (89.3)
Preprocedural TIMI 0-I (%)	191 (62.2)
Left main disease (%)	13 (4.1)
Multivessel disease (%)	200 (63.1)
Type of PCI	
Stent implantation (%)	272 (85.1)
Drug-eluting stents (%)	228 (71.3)
Bare metal stents (%)	44 (13.8)
Balloon angioplasty (%)	16 (5.0)
Others (%)	32 (10.0)
Postprocedural TIMI II-III (%)	298 (97.1)

**Territories of revascularization**	
**LMCA PCI (%)**	17 (5.4)
**LAD PCI (%)**	206 (65.2)
**LCX PCI (%)**	113 (35.8)
**RCA PCI (%)**	160 (50.6)
**PCI results**	
**Successful PCI**	307 (95.9)
**Suboptimal or failed PCI**	13 (4.1)

Values are presented number and percentage.

ACC, American College of Cardiology; AHA, American Heart Association; GPIIb/IIIa, glycoprotein IIb/IIIa complex; LAD, left anterior descending coronary artery; LCX, left circumflex coronary artery; LMCA, left main coronary artery; PCI, percutaneous coronary intervention; RCA, right coronary artery; TIMI, Thrombolysis in Myocardial Infarction.
